# Supplementary material for: Viral metagenomic investigation of two Caribbean echinoderms, Diadema antillarum (Echinoidea) and Holothuria floridana (Holothuria)
Source: PeerJ. 2024 Nov 26;12:e18321. doi: 10.7717/peerj.18321 (PMC11606319; doi:10.7717/peerj.18321)
Supplement: Supplemental Information 1 [file peerj-12-18321-s001.docx]

**Supplemental Table 1:** **Characteristics of transcriptomes prepared from *D. antillarum* used in viral query.**

| **Library** | **Reads** | **Tissue Type** | **Animal Condition** | **Site** | **Collection Date** | **NCBI Accession Number** |
| --- | --- | --- | --- | --- | --- | --- |
| tDaCF9 | 2167513 | Coelomic Fluid | Grossly Normal at Affected Site | Long Point, St John | 22-Apr-22 | SRR22260777 |
| tDaCF10 | 216188 | Coelomic Fluid | Grossly Normal at Affected Site | Long Point, St John | 22-Apr-22 | SRR22260776 |
| tDaCF13 | 1995447 | Coelomic Fluid | Reference | Pope Point, St John | 21-Apr-22 | SRR22260775 |
| tDaCF14 | 3095556 | Coelomic Fluid | Reference | Pope Point, St John | 21-Apr-22 | SRR22260774 |
| tDaCF17 | 423222 | Coelomic Fluid | Abnormal | Long Point, St John | 22-Apr-22 | SRR22260773 |
| tDaCF18 | 651867 | Coelomic Fluid | Abnormal | Long Point, St John | 22-Apr-22 | SRR22260772 |
| tDaC4ABW | 533173 | Body Wall | Reference | Tide Pools, Saba | 7-Apr-22 | SRR22260771 |
| tDaC5ABW | 334597 | Body Wall | Reference | Tide Pools, Saba | 7-Apr-22 | SRR22260770 |
| tDaC1ABW | 431190 | Body Wall | Grossly Normal at Affected Site | Diadema City, Saba | 7-Apr-22 | SRR29264044 |
| tDAC2ABW | 280235 | Body Wall | Grossly Normal at Affected Site | Diadema City, Saba | 7-Apr-22 | SRR29264045 |
