# Supplementary material for: Viral metagenomic investigation of two Caribbean echinoderms, Diadema antillarum (Echinoidea) and Holothuria floridana (Holothuria)
Source: PeerJ. 2024 Nov 26;12:e18321. doi: 10.7717/peerj.18321 (PMC11606319; doi:10.7717/peerj.18321)
Supplement: Supplemental Information 2 — Assembly algorithm precedes contig number (T, Trinity; M, metaSPAdes). E-values (E), ∗∗∗ < 1050, ∗∗1050 − 1030, ∗ > 1030 [file peerj-12-18321-s002.docx]

**Supplemental Table 2: BLAST hits of *Pisoniviricetes* viral contigs in *Holothuria floridana* viral metagenomes against the nonredundant and transcriptome shotgun assembly (TSA) archives at NCBI**. Assembly algorithm precedes contig number (T = Trinity; M = metaSPAdes). E-values (E): ***<10^50^, **10^50^-10^30^, *>10^30^

| **Contig** | **Length (nt)** | **Closest Blastx match to nr database** | **Accession** | **% ID** | **Cov. (%)** | **E** | **Host** | **Closest tBlastx match to TSA** | **Accession** | **% ID** | **Cov. (%)** | **E** |
| --- | --- | --- | --- | --- | --- | --- | --- | --- | --- | --- | --- | --- |
| T_DN23041 | 759 | Picornavirales sp. | QRG24242.1 | 64 | 59 | *** | *Pisaster ochraceus* | *Holothuria scabra* | GHHS01307730.1 | 56 | 65 | *** |
| T_DN1167 | 709 | Salisharnavirus sp. | ULG00075.1 | 57 | 48 | ** | River Sediment | *Apostichopus japonicus* | GFXQ02029053.1 | 57 | 42 | ** |
| T_DN13959 | 917 | Picornavirales N_OV_064 | ASG92536.1 | 39 | 96 | *** | Estuarine Water | *Patiria miniata* | GHJN010909143.1 | 42 | 56 | * |
| T_DN1454 | 546 | Picornaviridae sp. | URG14985.1 | 37 | 82 | * | River Sediment | No Hits |  |  |  |  |
| T_DN2641 | 1778 | Beihai paphia shell virus 2 | YP_009333343.1 | 39 | 37 | ** | Blue Swimmer Crab | *Eupentacta fraudtrix* | GHCL02047841.1 | 58 | 24 | * |
| T_DN27102 | 612 | Wenzhou picorna-like virus 8 | APG76682.1 | 51 | 71 | ** | Bivalve | *Patiria miniata* | GHJN011435149.1 | 67 | 51 | * |
| T_DN283 | 504 | Aurantiochytrium single-stranded RNA virus 01 | YP_398835.1 | 46 | 60 | * | *Schizochytrium* sp. | *Holothuria glabberina* | GIVL01267611.1 | 49 | 51 | * |
| T_DN30121 | 712 | Beihai picorna-like virus 91 | YP_009333596.1 | 42 | 83 | ** | Hermit Crab | No Hits |  |  |  |  |
| T_DN362 | 759 | Picornavirales N_OV_064 | ASG92536.1 | 43 | 71 | ** | Estuarine Water | No Hits |  |  |  |  |
| T_DN423 | 900 | Rudphi virus 5 | YP_009553173.1 | 41 | 89 | *** | *Ruditapes philippinarum* | No Hits |  |  |  |  |
| T_DN43 | 570 | Beihai picorna-like virus 91 | YP_009333596.1 | 53 | 80 | ** | Hermit Crab | *Apostichopus japonicus* | GHDI01331250.1 | 43 | 56 | * |
| T_DN4683 | 925 | Pycnopodia helianthoides associated picornavirus 2 | DAZ87485.1 | 60 | 92 | *** | Pycnopodia helianthoides | *Mesocentrotus franciscanus* | GHJZ01044897.1 | 47 | 45 |  |
| T_DN5059 | 630 | Beihai picorna-like virus 14 | YP_009333555.1 | 41 | 72 | * | Sea anemones | No Hits |  |  |  |  |
| T_DN643 | 703 | Beihai picorna-like virus 114 | YP_009333589.1 | 40 | 54 | * | Octopus | No Hits |  |  |  |  |
| T_DN7134 | 556 | Aurantiochytrium single-stranded RNA virus 01 | YP_398835.1 | 38 | 80 | * | Schizochytrium sp. | No Hits |  |  |  |  |
| M_NODE_2571 | 536 | Picornavirales Q_sR_OV_01 | ASG92550.1 | 60 | 78 | *** | Estuarine Water | *Apostichopus japonicus* | GFXQ02053930.1 | 37 | 72 |  |
| M_NODE_1047 | 661 | Picornavirales sp. | QRG24242.1 | 63 | 79 | *** | *Pisaster ochraceus* | *Holothuria scabra* | GHHS01307730.1 | 56 | 77 |  |
| M_NODE_1701 | 591 | Marine RNA virus PAL473 | YP_009230124.1 | 54 | 90 | *** | Antarctic Seawater | *Patiria miniata* | GHJN010932954.1 | 45 | 63 |  |
| M_NODE_3249 | 508 | *Aurantiochytrium* single-stranded RNA virus 01 | YP_392465.1 | 37 | 89 | * | *Schizochytrium* sp. | No Hits |  |  |  |  |
| M_NODE_2554 | 537 | Beihai picorna-like virus 114 | YP_009333589.1 | 53 | 42 | * | Octopus | No Hits |  |  |  |  |
| M_NODE_889 | 687 | Beihai picorna-like virus 91 | APG78010.1 | 45 | 66 | ** | Sesarmid Crab | No Hits |  |  |  |  |
| M_NODE_3044 | 516 | Beihei Picorna Virus 115 | YP_009333564.1 | 69 | 90 | *** | Sea anemones | No Hits |  |  |  |  |
| M_NODE_1602 | 600 | Fish-associated picorna-like virus 2 | WAQ80626.1 | 43 | 75 | * | Fish intestinal contents | No Hits |  |  |  |  |
| M_NODE_390 | 815 | Freshwater macrophyte associated picorna-like virus 8 | UQZ09590.1 | 39 | 76 | ** | Freshwater macrophyte | *Holothuria scabra* | GHHS01307730.1 | 37 | 79 | ** |
| M_NODE_2733 | 529 | Marine RNA virus PAL473 | YP_009230125.1 | 45 | 86 | * | Antarctic Seawater | No Hits |  |  |  |  |
| M_NODE_2506 | 539 | Picornavirales N_OV_064 | ASG92536.1 | 50 | 79 | ** | Estuarine Water | No Hits |  |  |  |  |
| M_NODE_3001 | 518 | Wenzhou picorna-like virus 8 | APG76682.1 | 47 | 68 | * | Bivalve | *Patiria miniata* | GHJN011435149.1 | 56 | 44 | * |
